# Effect of Support on the Activity of Ag-based Catalysts for Formaldehyde Oxidation

**DOI:** 10.1038/srep12950

**Published:** 2015-08-11

**Authors:** Jianghao Zhang, Yaobin Li, Yan Zhang, Min Chen, Lian Wang, Changbin Zhang, Hong He

**Affiliations:** 1 State Key Joint Laboratory of Environment Simulation and Pollution Control, Research Center for Eco-Environmental Sciences, Chinese Academy of Sciences, Beijing, 100085, China

## Abstract

Ag-based catalysts with different supports (TiO_2_, Al_2_O_3_ and CeO_2_) were prepared by impregnation method and subsequently tested for the catalytic oxidation of formaldehyde (HCHO) at low temperature. The Ag/TiO_2_ catalyst showed the distinctive catalytic performance, achieving the complete HCHO conversion at around 95 °C. In contrast, the Ag/Al_2_O_3_ and Ag/CeO_2_ catalysts displayed much lower activity and the 100% conversion was reached at 110 °C and higher than 125 °C, respectively. The Ag-based catalysts were next characterized by several methods. The characterization results revealed that supports have the dramatic influence on the Ag particle sizes and dispersion. Kinetic tests showed that the Ag based catalyst on the TiO_2_, Al_2_O_3_ or CeO_2_ supports have the similar apparent activation energy of 65 kJ mol^−1^, indicating that the catalytic mechanism keep immutability over these three catalysts. Therefore, Ag particle size and dispersion was confirmed to be the main factor affecting the catalytic performance for HCHO oxidation. The Ag/TiO_2_ catalyst has the highest Ag dispersion and the smallest Ag particle size, accordingly presenting the best catalytic performance for HCHO oxidation.

Formaldehyde (HCHO) is one of the major contaminants for the indoor air pollution and is detrimental to human’s health^1^. Long-term exposure to HCHO can cause irritation in eye, throat^2^, spatial memory deficits^3^, severe allergic reactions^4^ and even cancer^5^. Due to the growing concern for this hazard, enormous efforts have been made to remove the indoor HCHO.

Among the numerous kinds of methods for HCHO elimination, the catalytic oxidation has been proved to be a promising way to control HCHO pollution^6^. A certain kinds of catalysts have shown highly catalytic activity for HCHO oxidation. The supported noble metal catalysts such as alkali-metal-doped Na-Pt/TiO_2_^7^, Pt/MnO_x_-CeO_2_^8^, Na-promoted Pd/TiO_2_^9^ and Au/CeO_2_^10^ catalysts could convert HCHO to harmless CO_2_ and H_2_O at ambient temperature even at high space velocity. However, the high price of Pt, Au, Pd blocks their wide application and promotes the flourishing studies of supported Ag-based catalysts^11^,^12^,^13^,^14^,^15^,^16^,^17^ which has much lower price but shows the considerable efficiency for HCHO oxidation in low temperature. Qu *et al.*^14^ prepared Ag/SBA-15 by post-grafting method and it displayed 100 °C as the complete conversion temperature. A recent study reported that 1.7% K-Ag/Co_3_O_4_ could catalyze the oxidation of HCHO with 100% conversion at 70 °C^18^.

The activities of supported Ag catalysts are dramatically influenced by the supports, which have been attributed to the different states of sliver species on the supports^13^, diverse contents of the subsurface oxygen^19^, various oxygen storage and redox capacities^20^, etc. Ma *et al.*^12^ prepared Ag/CeO_2_ catalysts which exhibited distinct activities when the supports CeO_2_ were of diversity in morphology led by different synthesized methods. Chen *et al.*^13^ observed the dramatic variation in distribution of Ag nanoparticle diameters, redox capacities and HCHO desorption properties when Ag was loaded on the supports such as TiO_2_, MCM-41, SBA-15, etc. Shi *et al.*^17^ studied the support effects of Mn-Ce oxides and H-ZSM-5 on Ag catalysts and found that Ag/MnO_x_-CeO_2_ showed much higher activity since Mn-Ce oxides could accelerate the partial oxidation of HCHO into HCOO^−^.

Due to the enormous distinction in activity displayed in supported Ag catalysts, it is of great significance to reveal the internal discipline of support effect. TiO_2_, γ-Al_2_O_3_ and CeO_2_ have been proved to be stable support for several catalytic reactions such as HCHO oxidation^12^,^21^,^22^,^23^, NO_x_ selective degeneration^24^,^25^,^26^,^27^, formic acid decomposition^28^, hydrogen production^29^, etc. Therefore, in this work, we prepared the Ag-based catalysts with supports of TiO_2_, γ-Al_2_O_3_ and CeO_2_ by impregnation method and then compared their performance for the catalytic oxidation of HCHO at low temperature. The dramatic difference about the catalytic activity was clearly observed on the three catalysts. Ag/TiO_2_ exhibited the best activity, archiving 100% conversion of 110 ppm HCHO at around 95 °C with a gas hourly space velocity of 100 000 mL (g_cat_·h)^−1^. The Brunauer-Emmett-Teller (BET), X-ray diffraction (XRD), High-resolution transmission electron microscope (HRTEM), UV-vis diffuse reflectance spectroscopy (DRS), X-ray photoelectron spectroscopy (XPS), Temperature-programmed reduction (TPR) were next measured to characterize the catalysts. Based on the results, the internal rules influencing the activity of Ag based catalysts were discussed and elucidated.

## Results and Discussion

### Performance Test of HCHO Catalytic Oxidation

**Figure 1:**
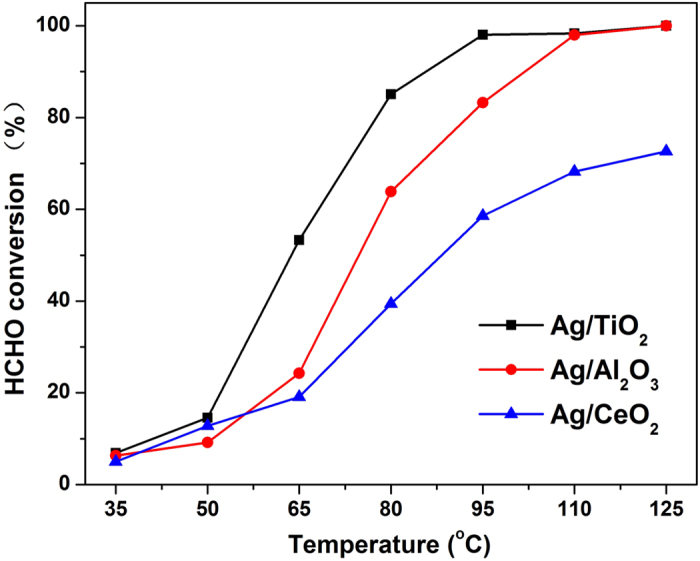
HCHO conversion over Ag/TiO_2_, Ag/Al_2_O_3_ and Ag/CeO_2_ samples. Reaction condition: 110 ppm of HCHO, 20% O_2_, N_2_ balance, GHSV = 100 000 mL (g_cat_·h)^−1^.

Figure 1 displays the HCHO conversions as a function of temperature over the Ag/TiO_2_, Ag/Al_2_O_3_ and Ag/CeO_2_ catalysts at a GHSV of 100 000 mL (g_cat_·h)^−1^ with the inlet HCHO concentration of 110 ppm. The temperature dependence of conversion rates were dramatically affected by the supports, and the catalytic activities followed the order of Ag/TiO_2_ > Ag/Al_2_O_3_ > Ag/CeO_2_. The Ag/TiO_2_ catalyst showed the highest HCHO conversion rate at each testing temperature point and reached the 100% conversion at around 95 °C. The Ag/Al_2_O_3_ sample exhibited much lower activity with complete conversion at 110 °C, and the HCHO over Ag/CeO_2_ could not be entirely decomposed even when the temperature was ramped to 125 °C.

We also prepared the Ag/TiO_2_ with different ratio as 4, 6, 10, 12 wt% and their activities are shown in Figure S1 and S2 (supporting information). The activity as a function of content displayed a typical volcano curve and the best activity emerges when the content was 8 and 10 wt%, which is the reason for choosing 8 wt% to implement this research. The pure supports (TiO_2_, Al_2_O_3_ and CeO_2_) showed no activity for HCHO oxidation in the testing temperature range (35–125 °C) (data not shown). Therefore, the loaded Ag should be mainly responsible for the huge distinction in catalytic performance. Similar support effect was also found among the Pt-based catalysts with support as TiO_2_, SiO_2_, Ce & Zr oxides for HCHO oxidation mainly due to the Pt dispersion variation on different supports^30^. A theoretical study on Au-based catalysts showed that different supports could affect the charge transfer from Au to support materials, therefore showing the different activity for glucose oxidation^31^. Due to the numerous explanations by many studies, investigating the factors that lead to such diversity of catalytic performances from the same Ag active species would be in considerable significance.

### Structure Analysis

To check the possibility of influence from surface physical property, the specific surface areas (BET), average pore diameters, and total pore volumes of the three samples were first measured, subsequently the specific reaction rate (R_s_) at 80 °C were calculated. As shown in Table 1, the Ag/TiO_2_ catalyst had the lowest surface area but presented the highest catalytic activity, leading to its largest R_s_ of 2.18 nmol s^−1^ m^−2^ at 80 °C among the three samples. The Ag/Al_2_O_3_ displayed the higher surface area, apparent activity and also R_s_ than the Ag/CeO_2_ catalyst. These results indicate that the physical properties have the limited influence on catalytic performance.

**Figure 2:**
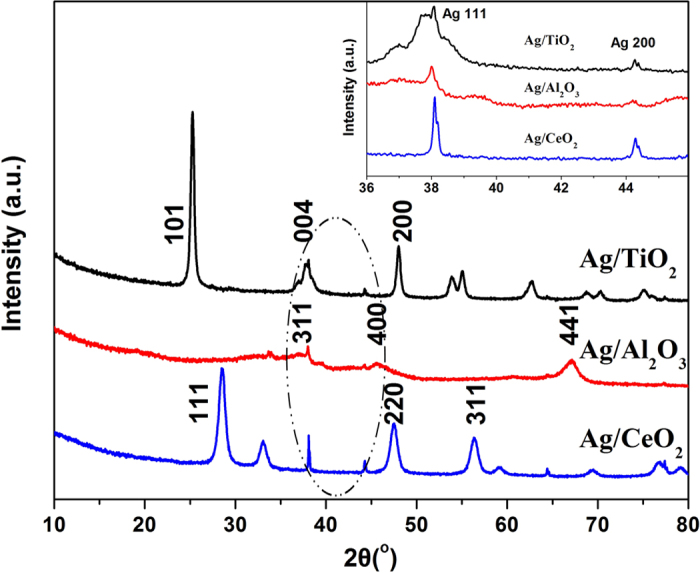
XRD patterns of the Ag/TiO_2_, Ag/Al_2_O_3_ and Ag/CeO_2_ catalysts (inset: magnification of the scale from 36° to 46°).

XRD measurement was next carried out to investigate the crystallographic structures. As shown in Fig. 2, the intensive peaks in the characteristic locations of TiO_2_ with anatase structure (JCPDS 71-1167) and CeO_2_ (JCPDS 43-1002) indicated the dominating content of the respective supports. Unlike the former two supports, γ-Al_2_O_3_ (JCPDS 74-2206) was formed with amorphous structure, leading to the poor intensity in the pattern. In addition, the peaks of metallic silver could be also detected, which could be indexed to face-centered cubic crystal structures, while no diffraction due to silver oxide was observed. Two peaks (shown in the magnified inset graph) located at 38.1° and 44.3° could be assigned to diffraction line of the (111) and (200) planes of the metallic silver crystal, respectively (JCPDS 87-0597). The Ag (111) peak at 38.1° in Ag/TiO_2_ pattern was overlapped with the TiO_2_ (anatase) peaks of (103), (004), (112) facets. It was still observed that the peak intensities corresponding to silver were much different over three samples. Noting that the intensity of XRD pattern reflects the crystallinity and the crystal size, we therefore speculate that the Ag particle sizes over each sample are much different among samples. The largest Ag average particle size might emerge on Ag/CeO_2_ which displays the most intensive peak for Ag crystal and the smallest Ag average particle size should exist on Ag/TiO_2_ sample.

**Figure 3:**
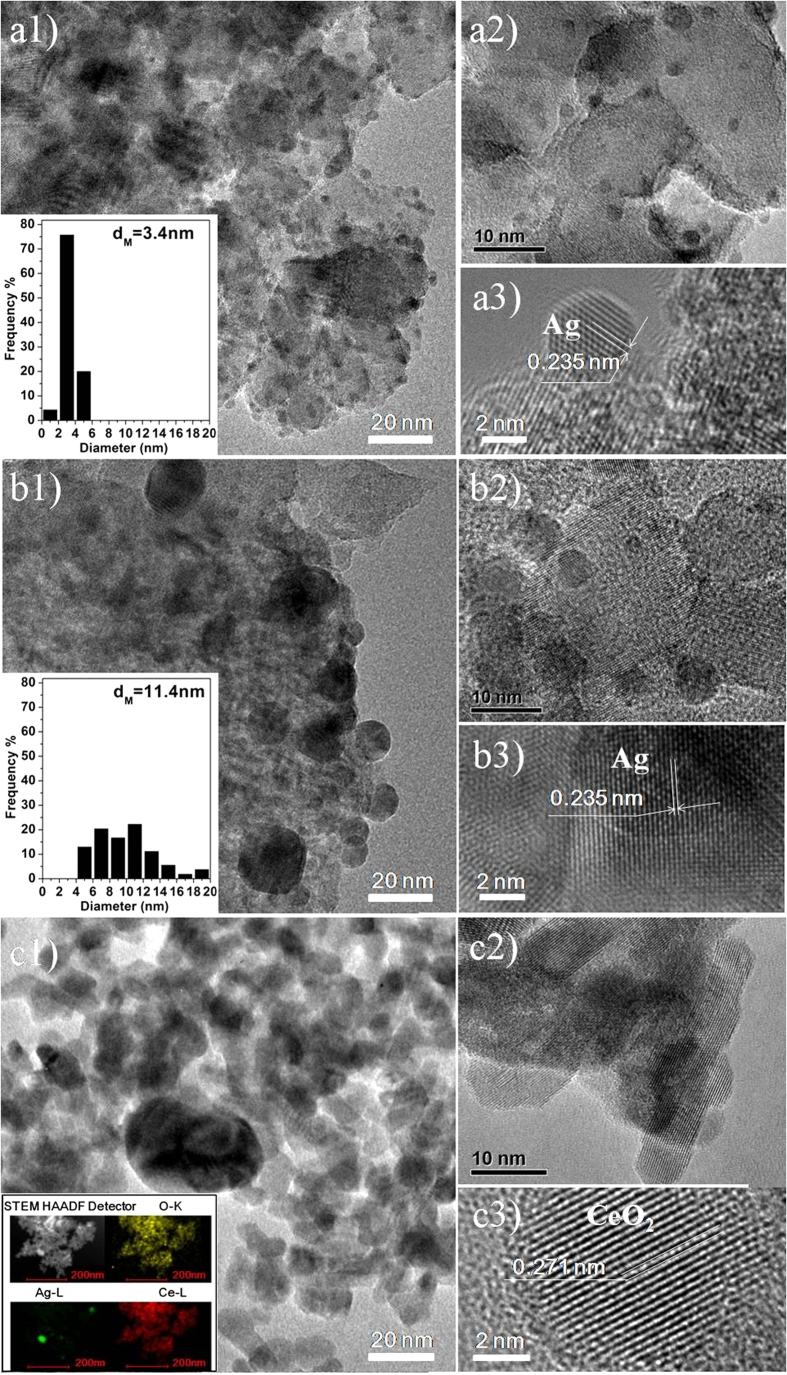
High-resolution TEM images of the catalysts: Ag/TiO_2_ (**a**) Ag/Al_2_O_3_ (**b**) and Ag/CeO_2_ (**c**) 1,2,3 refer to different magnifications. Inset: statistic results of mean particle diameter by measuring ~100 Ag particles for Ag/TiO_2_ and Ag/Al_2_O_3_, maps of the Ag (green), oxygen (yellow), cerium (red) for Ag/CeO_2_.

We next implemented the HR-TEM measurement to investigate the states and particles size distribution of Ag over three catalysts. The images shown in Fig. 3 are representative for the entire surface of the respective samples. As shown in the inset histogram of statistic results from 100 particles and the summary in Table 2, the Ag particles on Ag/TiO_2_ displayed homogeneous distribution with a mean diameter of 3.4 nm, being close to the previous result^13^. In contrast, the Ag particles on Ag/Al_2_O_3_ (Fig. 3b) grew larger with the average size of 11.3 nm and the distribution was also not as uniform as that on Ag/TiO_2_. Being different with the former two samples, the Ag particle over Ag/CeO_2_ (Fig. 3c) was undistinguishable with that of support CeO_2_ on HR-TEM image. Therefore, mapping was next carried out to find out the morphology and location of Ag species on CeO_2_. It could be seen from the mapping (insert in Fig. 3c1) that the supported Ag was poorly dispersed on CeO_2_ with large particle size (>30 nm), being consistent with the previous study^32^ which reported that a few large Ag particles on CeO_2_ could even reach to 400 nm. Taking into account of the incomparability between bright field and mapping images, we did not make statistic for Ag/CeO_2_. However, the HR-TEM images still approved the deduction from the XRD results that the Ag on CeO_2_ was rather poorly dispersed. It is also confirmed that the Ag particle size on TiO_2_ was the smallest among the three kinds of catalysts.

### Chemical Characterization

**Figure 4:**
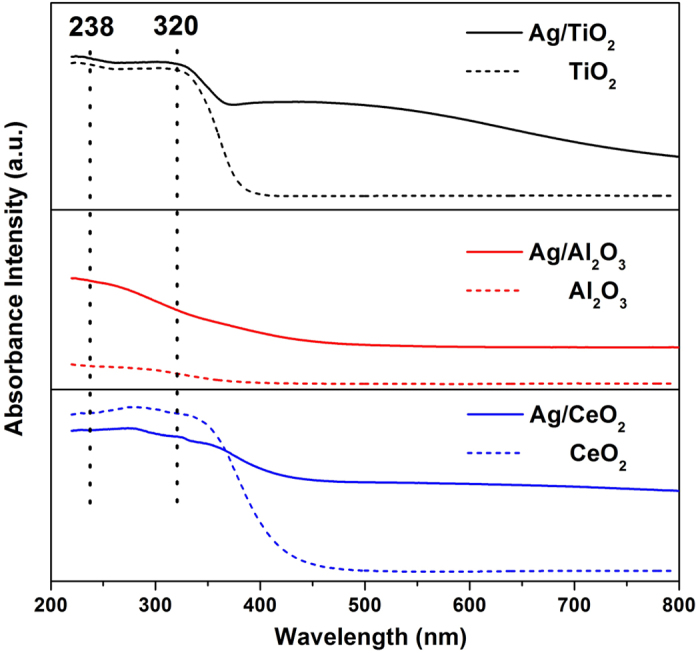
Diffuse-reflectance of UV-Vis spectra of the catalysts and the supports. (Straight line: the catalysts, Dash line: the pure supports).

UV-Vis measurement was carried out to discover the details of the state of supported Ag. Figure 4 depicts the UV-vis spectra of both the catalysts and the supports. According to the previous literatures^13^,^33^, the absorbance below λ = 238 nm should be assigned to the 4d^10^ to 4d^9^5s^1^ transition of highly dispersed Ag^+^ ions; the band at 238–320 nm is attributed to small Ag_n_^δ+^ clusters, and the absorbance above 320 nm is due to the existence of the metallic Ag^0^. From the comparison between the spectra of the Ag loaded catalysts and pure supports, it was revealed that the absorbance in the whole scale generally were more intensive after Ag was loaded. The bands could be deemed as a description of distribution of different Ag species^33^. It could be speculated that the Ag over the catalysts mainly presented the metallic crystal status since the band area in Ag^0^ region was larger than others. Another interesting observation was that the peak intensities in 220–350 nm were enhanced over Ag/TiO_2_ and Ag/Al_2_O_3_ but diminished over Ag/CeO_2_ after Ag loading. One speculation was that a few large Ag particles on CeO_2_, which presented the Ag^0^ state, blocked the absorbing of UV but enhanced the absorbance of visible light.

**Figure 5:**
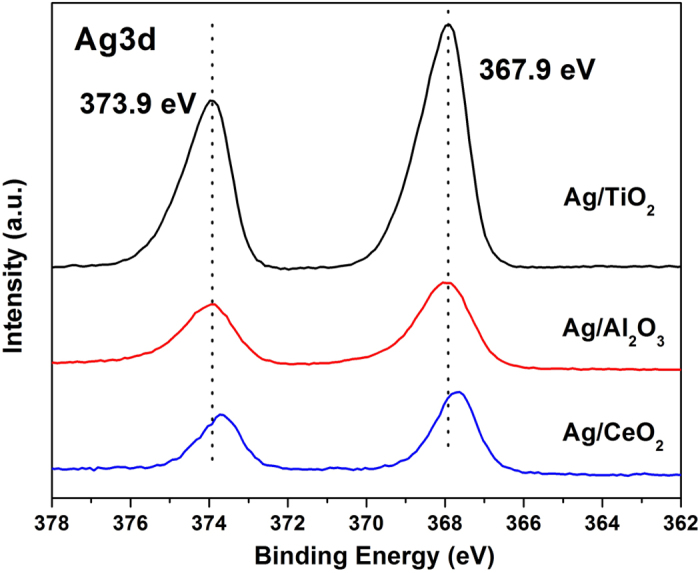
Ag3d XPS results of Ag/TiO_2_, Ag/Al_2_O_3_ and Ag/CeO_2_ catalysts.

To further check the electronic state of surface Ag, the XPS spectra were measured. Figure 5 shows the Ag3d spectra of the three catalysts. The Ag/TiO_2_ showed two peaks at binding energies of 367.9 eV (Ag3d_5/2_) and 373.9 eV (Ag3d_3/2_), which were close to those expected for metallic silver (368.0 eV and 374.0 eV)^34^, indicating that the Ag on TiO_2_ was mainly in the metallic state, being consistent with the XRD and UV-vis results. This finding could be corroborated by silver oxide’s thermal decomposition study^35^, showing that the Ag_2_O will decompose to metallic silver with the thermal treatment at 400 °C, which is lower than our calcination temperature of 450 °C. Since different support would lead to different and complex electronic environment, the Ag3d peaks showed a slight shift over each catalysts, which could be also observed by many documents^12^,^18^,^36^.

It was most noted that the intensities of Ag peaks for three catalysts displayed dramatic distinction. As shown in Table 2, the peak areas of Ag3d_5/2_ for Ag/TiO_2_ and Ag/Al_2_O_3_ were 3.29 and 1.34 times of that for Ag/CeO_2_ sample. The peak area is the function of the atom numbers of element when the XPS spectra are measured in the same condition for the same Ag element^37^. Thus, the peak area was exclusively related to the number of Ag atoms in the scanning volume. When the Ag was highly dispersed over support, there would be much Ag atoms exposed to the surface and emitted photoelectrons, consequently led to the high intensity of Ag3d spectra lines. Therefore, the XPS results showed that Ag species on TiO_2_ was well dispersed and the sequence of dispersion degree should be Ag/TiO_2_ > Ag/Al_2_O_3_ > Ag/CeO_2_, which is well consistent with the former characterization results.

One preceding work^38^ had reported the size effect over gold catalysts, which showed markedly enhanced catalytic activity for CO oxidation as the gold particles are smaller than 10 nm. Another study^39^ reported the remarkable size effect of ruthenium particles also for the catalytic oxidation of CO. Our study is also prone to the size effect as the indispensible factor that lead to the dramatic distinction in the catalytic activities for HCHO oxidation. The particle size on of Ag on TiO_2_ is the smallest among the three supports, consequently contribute to the considerable enhancement of the activity.

**Figure 6:**
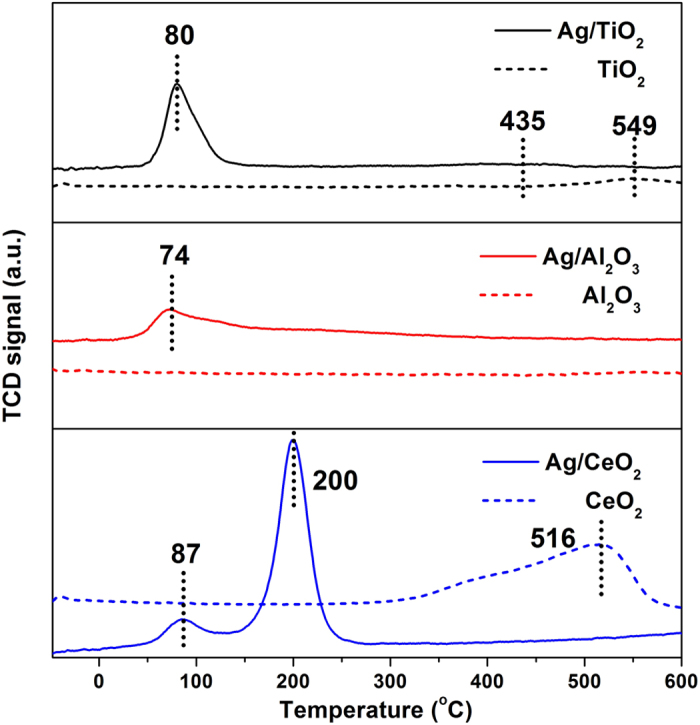
H_2_-TPR profiles of the catalysts and supports.

H_2_-TPR experiments were next performed to investigate the reducibility of three samples. Figure 6 presented the H_2_-TPR profiles of both the Ag catalysts and the corresponding supports. The pattern of Ag/TiO_2_ sample showed two reduction peaks at around 80 °C and 435 °C. Since the reduction peak of Ag_2_O powder normally centered at above 100 °C^40^, the peak located at 80 °C should be mainly ascribed to reduction of oxygen species absorbed on the dispersed Ag surface. The peak at 435 °C should be due to the reduction of surface capping oxygen of TiO_2_. In contrast, the pure TiO_2_ presented a single reduction peak at 549 °C, indicating that the Ag presence facilitated the reduction of TiO_2_ surface oxygen species.

Different from pure TiO_2_ sample, no reduction peak was observed on pure γ-Al_2_O_3_ in the examined temperature range (−50–600 °C). Consequently, the Ag/γ-Al_2_O_3_ only showed one peak at 74 °C, which should be exclusively related to the supported Ag species. As for the Ag/CeO_2_, a low temperature reduction at 87 °C also appeared, similar to other two samples. The TPR profile of pure CeO_2_ is characterized by wide peak in the region of 300 °C to 600 °C, which is typical for the reduction of surface capping oxygen species on CeO_2_^41^. The Ag loading also induced the shift of the CeO_2_ reduction to low temperature at around 200 °C, which is consistent with the previous study^12^.

Three Ag supported catalysts all displayed the similar reduction peaks at around 80 °C, indicating the similar oxygen mobility over these catalysts. The difference lied on the H_2_ consumption amount, which were calculated from the profiles and presented in Table 2. The Ag/TiO_2_ catalyst had the largest H_2_ consumption amount with 166.9 μmol g^−1^. The Ag/Al_2_O_3_ and Ag/CeO_2_ presented the amounts of 97.1 μmol g^−1^ and 69.9 μmol g^−1^, respectively. This sequence was well aligned with the activity order, indicating the catalytic performance should be closely related to the amount of active oxygen species. The different H_2_ consumption amounts might be originated from the disparity of Ag particles over these three catalysts. Ghosh *et al.*^42^ reported that the supported metallic Ag nanoparticles could activate the molecular oxygen and then catalyze the oxidation reaction. The Ag/TiO_2_ catalyst possessed the best Ag dispersion, which is beneficial to oxygen activation and then led to the most sufficiency of active oxygen species, consequently exhibited the best catalytic activity.

H_2_-O_2_ titration method is widely utilized to determine the dispersion of Ag on γ-Al_2_O_3_^43^. However, this method needs the 170 °C to perform the measurement of Ag dispersion, therefore not suitable to the Ag/CeO_2_ catalyst which displays the mixed reduction of both oxygen species absorbed on Ag surface and CeO_2_ support at this temperature. In contrast, the intensity of reduction peak at around 80 °C in H_2_-TPR profiles was proportional to the amount of oxygen species absorbed on Ag surface, therefore it may accurately respond to the Ag dispersion on each support. We then calculated the Ag particle sizes based on the H_2_ consumption amounts and the results are given in Table 2. The diameters of Ag particles are 5.5, 9.5 and 13.1 nm on TiO_2_, Al_2_O_3_ and CeO_2_, respectively. Although there is slight deviation between the statistics from HR-TEM and calculation by H_2_ consumption^44^, the sequences of Ag particle sizes from both methods are the same for three catalysts.

**Figure 7:**
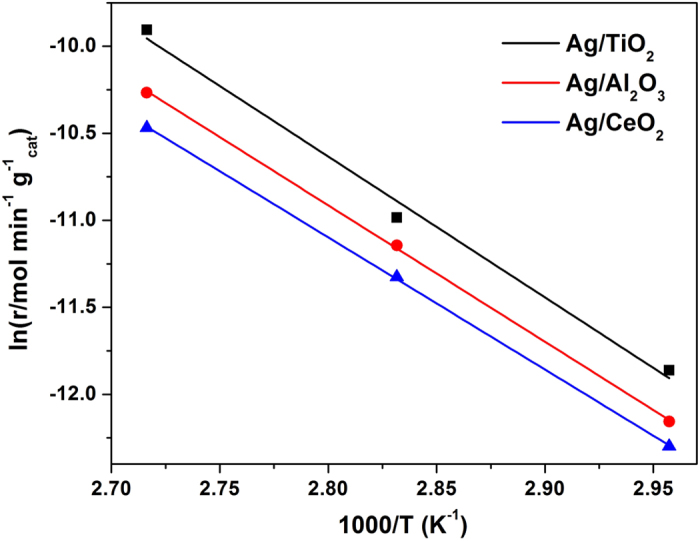
Arrhenius plots of HCHO oxidation on the catalysts.

The high dispersion of Ag species on support enhanced the amount of exposed active centers and consequently contributed to high activity. However, there is another possibility for the size effect that the reaction mechanism might be changed when the Ag particle size dropped^45^. Therefore, we next implemented the kinetic measurement to investigate the change of reaction mechanism. Figure 7 displays the Arrhenius plots of the three catalysts, which were tested at high GHSV condition to control the HCHO conversion below 15%. We then calculated the apparent activation energies and the results are presented in Table 2. All the three samples display almost the same activation energy with 67 kJ mol^−1^, 65 kJ mol^−1^, 63 kJ mol^−1^ for Ag/TiO_2_, Ag/Al_2_O_3_ and Ag/CeO_2_ catalysts, respectively. The turnover frequency at 95 °C is also calculated based on the amount of Ag sites calculated from the H_2_-TPR results. The data in Table 2 revealed that Ag/TiO_2_, Ag/Al_2_O_3_ and Ag/CeO_2_ had the similar TOFs of 0.0050 s^−1^, 0.0060 s^−1^, 0.0068 s^−1^, respectively. These results indicate that the differences of both Ag particle sizes and support types did not clearly influence the reaction mechanism of HCHO oxidation, but mainly changed the pre-exponential factor of the rate law^46^. Thus, we further confirmed that the dispersion and particle size of supported Ag species was the main factor affecting the catalytic activity.

In summary, the Ag supported on TiO_2_, Al_2_O_3_ and CeO_2_ catalysts were prepared by impregnation method. The Ag/TiO_2_ catalyst showed the highest catalytic performance among three catalysts, achieving the complete HCHO conversion at around 95 °C. The Ag particle size was found to be the main factor influencing the catalytic performance of Ag supported catalysts for HCHO oxidation. Different supports could drastically affect the Ag particle size, consequently influence the catalytic activity. TiO_2_ was found to be the most suitable support for Ag to catalyze the oxidation of HCHO since it could induce the best dispersion and the smallest particle size of Ag species on surface. Despite of the considerable distinction in activities among three catalysts, the reaction mechanism of HCHO oxidation remained unchanged. It was also demonstrated that Ag/TiO_2_ catalyst may potentially be utilized to industrial application for HCHO oxidation.

## Methods

### Catalysts Preparation

The three kinds of samples (8 wt% Ag/support) were prepared by solution impregnation of each support at room temperature, using an aqueous solution of AgNO_3_. After stirring for 1 h, excess water was removed in a rotary evaporator at 30–50 °C under vacuum until dryness. Then, the samples were dried at 100 °C overnight and calcined at 450 °C with ramping rate of 5 °C min^−1^ in static air for 3 h.

The TiO_2_ with anatase structure and γ-Al_2_O_3_ powder were commercialized samples. The CeO_2_ support was prepared with the method similar to the previous documents^47^,^48^. In detail, 9 mmol cerium nitrate and 0.375 mol NaOH were dissolved in 30 ml and 50 ml of deionized water, respectively. After blending the two kinds of solution in a beaker, the mixture was stirred for 60 min and subsequently transferred into a Teflon-lined stainless autoclave at a temperature of 100 °C and held for 12 h. The fresh precipitates were thoroughly washed with deionized water and anhydrous ethanol. The solid obtained was dried at 60 °C in air for 24 h and calcined at 550 °C for 4 h in air.

### Catalyst Characterization

The structure parameter, pore size and specific surface area of the samples were obtained by the BET plot using a Quantachrome Quadrasorb SI-MP at −196 °C over the whole range of relative pressures. The pore size distribution was calculated by the desorption branch of the N_2_ adsorption isotherm using the BJH method. Before the N_2_ physisorption, the catalysts were degassed at 300 °C for 5 h. XRD patterns were measured on an X’Pert PRO MPD X-ray powder diffractionmeter with a Cu Kα (λ = 0.154056 nm) radiation operated at 40 kV and 40 mA. The 2θ angle ranged from 10° to 80° with a scan step of 0.02°.

HRTEM was performed on a FEI Tecnai G^2^ F20 electron microscope operating at 200 kV with a supplied software for automated electron tomography. Typically, a drop of the nanoparticle solution was dispensed onto a 3-mm carbon-coated copper grid. Excess solution was removed by an absorbent paper, and the sample was dried at room temperature. Average particle size and particle size distribution were sampled by ~100 Ag particles from randomly chosen areas of the TEM image. The mean particle diameter (d_M_) was calculated as the previous report^30^ by the formula: 
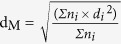
, where d_*i*_ is the particle size and n_*i*_ is the number of particles in size range between d_*i*_ + Δ and d_*i*_.

The UV-vis DRS of the catalysts and supports were recorded with a UV-2600 spectrophotometer. The spectra were recorded in the 220–800 nm wavelength region with a collection speed of 80 nm min^−1^ at ambient temperature. XPS profile was obtained by an AXIS Ultra system, equipped with an Al Kα radiation (*hv* = 1486.6 eV) with anode operated at 225 W and 15 kV. The binding energy values were calibrated by C 1 s peak (284.8 eV).

H_2_-TPR were carried out on Chemisorption Analyzer (AutoChem 2920) equipped with a TCD detector. After sweeping by air at 300 °C and declining down to 0 °C in the flow of Ar, 10% H_2_/Ar and air successfully flowed through the samples. Then the temperature ramped to −50 °C at the atmosphere of Ar. When the reference gas of 10% H_2_/Ar at a rate of 50 cm^3^ min^−1^ stabilized the TCD signal, the temperature started to ramp from −50 to 600 °C at a rate of 10 °C min^−1^. The H_2_ consumption was monitored by TCD after produced H_2_O removal. The dispersion (D) data of catalysts was determined by the H_2_ consumption amount. The Ag/O_2_/H_2_ stoichiometry was assumed as 2/1/2 and the particle size was calculated by the formula d (nm) = 1.34/D as the previous document^33^,^49^.

### Measurement of Catalytic Activity

The activity tests for the catalytic oxidation of HCHO over the catalysts (60 mg) were performed in a fixed-bed quartz flow reactor (i.d. = 4 mm) in an incubator. The catalysts were pressed in the same pressure and sieved to collect the portion of 40–60 mesh. Gaseous HCHO was generated by flowing nitrogen through the paraformaldehyde container in a water bath kept at 35 °C. The feed gas composition was 110 ppm HCHO, 20% O_2_ balanced by N_2_. The total flow rate was 100 mL min^−1^, corresponding to a gas hourly space velocity (GHSV) of 100000 mL (g_cat_·h)^−1^.

Kinetics measurement was implemented as the HCHO conversion being kept below 15%. Kinetics data were tested under the condition of HCHO concentration with 1400 ppm and GHSV with 476000 mL (g_cat_·h)^−1^ for Ag/TiO_2_, 341000 mL (g_cat_·h)^−1^ for Ag/Al_2_O_3_ and 302000 mL (g_cat_·h)^−1^ for Ag/CeO_2_ catalysts. The total flows all contained 20% O_2_ and N_2_ was the balance gas.

As the same with our previous activity evaluating instruments and methods^6^, the inlet and outlet gases were monitored by FTIR (Nicolet iS50) equipped with 2 m gas cell and a DTGS detector; resolution: 0.5 cm^−1^; OPD velocity: 0.4747 cm s^−1^. The collect region was 4000–600 cm^−1^ and the number of scans per spectrum was 16 times. HCHO and CO_2_ was measured by the peaks located at 2897 (C-H vibration) and 2350 cm^−1^ (O-C-O vibration), respectively. The HCHO and CO_2_ concentrations were quantified and calculated based on the peak area of CO_2_ at 2350 cm^−1^. Since no other carbon containing compounds except for CO_2_ were detected in the effluents for all tested catalysts, the conversion was calculated from a carbon balance that 1 mol of HCHO forms 1 mol of CO_2_.

## Additional Information

**How to cite this article**: Zhang, J. *et al.* Effect of Support on the Activity of Ag-based Catalysts for Formaldehyde Oxidation. *Sci. Rep.* **5**, 12950; doi: 10.1038/srep12950 (2015).

## Supplementary Material

Supplementary Information

## Figures and Tables

**Table 1:** Physical Parameter and Specific Reaction Rate (R_s_) at 80 °C of the Catalysts.

Catalyst	BET (m^2^/g)	pore diameter (nm)	pore volume (cm^3^/g)	R_s_ (nmol/s/m^2^)
Ag/TiO_2_	53.2	19.4	0.258	2.18
Ag/Al_2_O_3_	154.2	6.2	0.240	0.57
Ag/CeO_2_	113.7	15.1	0.430	0.47

**Table 2:** Relative Peak Area of Photoelectrons from Ag3d_5/2_, Ag Particle Size Results, H_2_ Consumption in H_2_-TPR, TOFs at 95 °C and Arrhenius Activation Energy of the Catalysts.

catalyst	peak area of Ag3d_5/2_^1^	particle size (nm)		H_2_ consumption in H_2_-TPR (μmol/g)	TOF at 95 °C(1/s)	activation energy (kJ/mol)
		d_M_^2^	d_C_^3^			
Ag/TiO_2_	3.29	3.4	5.5	166.9	0.0050	67
Ag/Al_2_O_3_	1.34	11.4	8.1	97.1	0.0060	65
Ag/CeO_2_	1.00	—	13.1	69.9	0.0068	63

^1^The area is a relative one when that of Ag/CeO_2_ is set as 1.00.

^2^Statistic mean particle size by HRTEM,

^3^Calculated particle size from dispersion results by H_2_-TPR.
