# Supplementary material for: Effect of Support on the Activity of Ag-based Catalysts for Formaldehyde Oxidation
Source: Sci Rep. 2015 Aug 11;5:12950. doi: 10.1038/srep12950 (PMC4531785; doi:10.1038/srep12950)
Supplement: Supplementary Information [file srep12950-s1.pdf]

Effect of Support to the Activity of Ag-based Catalysts for Formaldehyde Oxidation

Jianghao Zhang, Yaobin Li, Yan Zhang, Min Chen, Lian Wang, Changbin Zhang*, Hong He.

State Key Joint Laboratory of Environment Simulation and Pollution Control,

Research Center for Eco-Environmental Sciences, Chinese Academy of Sciences

Beijing, 100085, China.

*Corresponding author: Phone: 86-10-62849121; fax: 86-10-62849121; e-mail: cbzhang@rcees.ac.cn

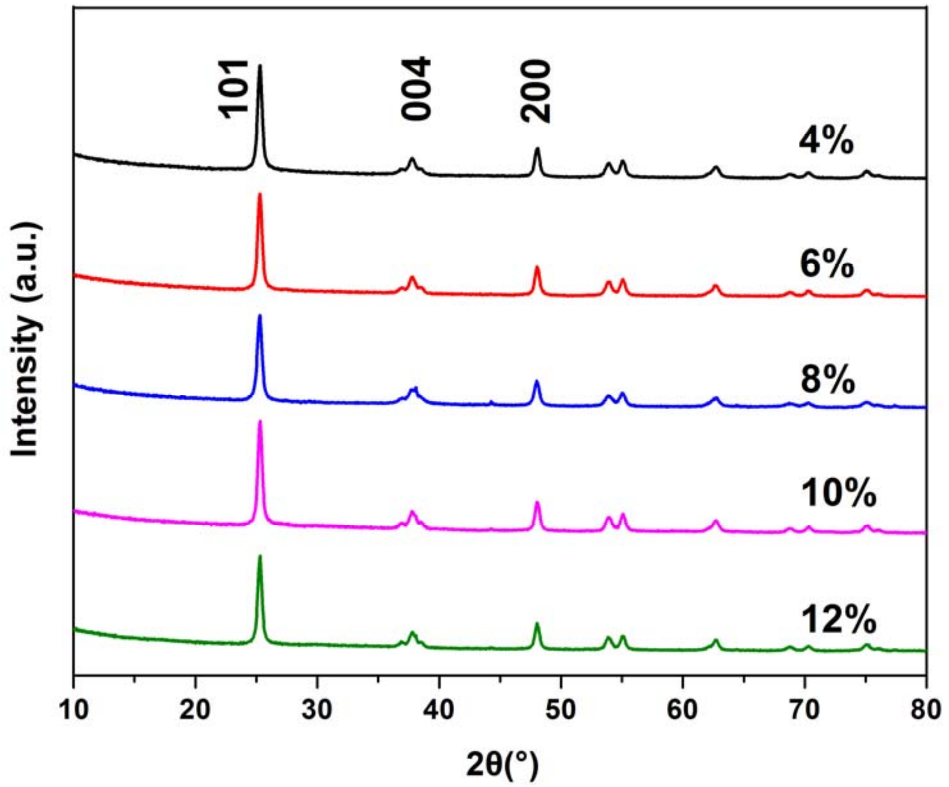

Figure S1. XRD patterns of the Ag/TiO_2 catalysts with Ag contents of 4, 6, 8, 10, 12 wt %.

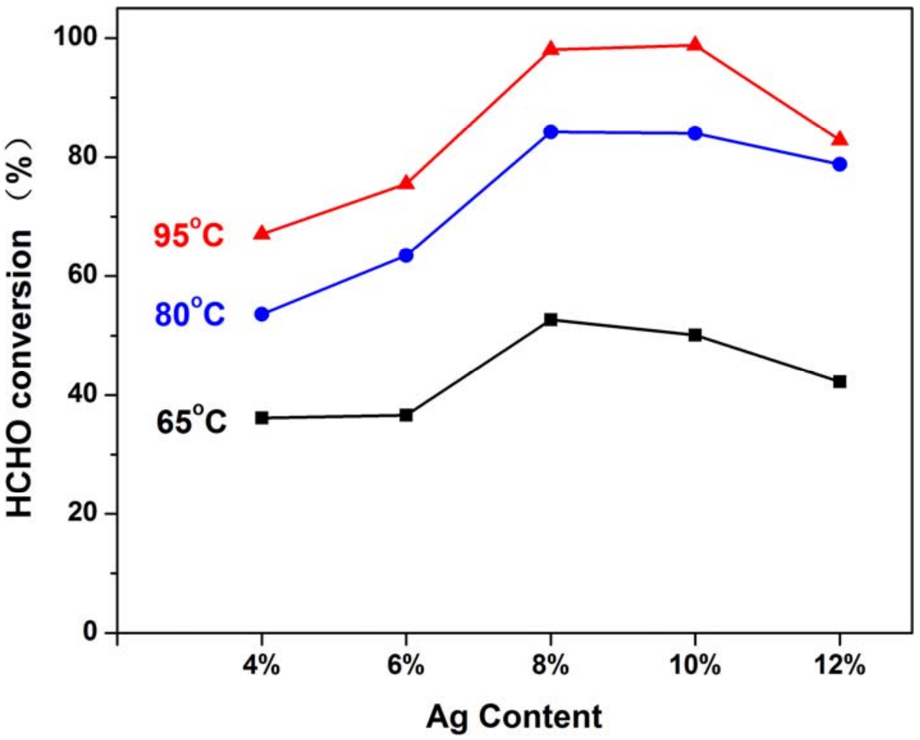

Figure S2. HCHO conversion over Ag/TiO_2 with Ag contents of 4, 6, 8, 10, 12 wt %. Reaction condition: 110 ppm of HCHO, 20% O_2, N_2 balance, GHSV=100 000 mL $(g_{cat}\cdot h)^{-1}$.
